# Combining clinical characteristics with CT radiomics to predict Ki67 expression level of small renal mass based on artificial intelligence algorithms

**DOI:** 10.3389/fonc.2025.1541143

**Published:** 2025-02-21

**Authors:** Junyi Lin, Yongyi Ou, Mingli Luo, Xuxuan Jiang, Shengren Cen, Guohua Zeng

**Affiliations:** ^1^ Department of Urology, The First Affiliated Hospital, Guangzhou Medical University, Guangzhou, Guangdong, China; ^2^ Guangdong Provincial Key Laboratory of Urology Diseases, Guangzhou Medical University, Guangzhou, Guangdong, China; ^3^ Guangdong Engineering Research Center of Urinary Minimally Invasive Surgery Robot and Intelligent Equipment, Guangzhou Medical University, Guangzhou, Guangdong, China; ^4^ Guangzhou Institute of Urology, Guangzhou Medical University, Guangzhou, Guangdong, China; ^5^ Department of Urology, Sun Yat-sen Memorial Hospital, Sun Yat-sen University, Guangzhou, Guangdong, China; ^6^ Department of Ophthalmology, Sun Yat-sen Memorial Hospital, Sun Yat-sen University, Guangzhou, Guangdong, China

**Keywords:** small renal mass, Ki67, CT, radiomics, artificial intelligence

## Abstract

**Background:**

Most small renal masses (SRMs) grow slowly and have good prognosis, but a portion of SRMs can also demonstrate aggressive characteristics, which can be explored by the proliferation-related marker Ki67.

**Methods:**

A total of 241 patients collected from the two centers were included in the study, of which 145 patients from the First Affiliated Hospital of Guangzhou Medical University were divided into training and validation cohort, while 96 patients from Sun Yat-sen Memorial Hospital were served as test cohort. To ensure the class balance of the outcome measures, the training cohort was oversampled, resulting in an increase of 77 cases in the minority class. After variables processing and feature selecting, optimal artificial intelligence-based model was constructed to predict the Ki67 expression level, and the model performance, interpretation and application development was performed.

**Results:**

The baseline characteristics of enrolled patients were described, and no statistically significant differences were found between two centers and cohorts, both before and after oversampling. The optimal model, regularized random forest, was constructed showing AUROC values of 0.802, 0.878, and 0.668, and balanced accuracy of 0.744, 0.808, and 0.679 in the oversampling training, validation, and test cohort, respectively. Model interpretation was performed, and a web application was built.

**Conclusions:**

An artificial intelligence-based predictive model for non-invasively assessing the Ki67 expression level of SRMs was developed, thus providing valuable reference for clinical decision-making in these patients.

## 1 Introduction

According to the guidelines of the American Society of Clinical Oncology (1), small renal masses (SRMs) are defined as renal tumors detected incidentally on imaging with diameter less than or equal to 4 cm, representing a group of tumors that include benign, indolent, and aggressive types. The standard treatment recommended by the guidelines is partial nephrectomy, with active surveillance or renal tumor biopsy considered in specific circumstances. The five-year cancer-specific survival probability for SRMs ranges from 95% to 100% (2), as most tumors exhibit an indolent natural progression with slow growth and minimal metastasis. However, a subset of SRMs can also demonstrate aggressive characteristics, which are associated with clear cell subtype, high grade, positive p53 expression, and high Ki67 expression (3). Clinical factors and conventional imaging often poorly predict this aggressive feature, while immunohistochemistry can explore the nature of tumor growth and invasiveness by detecting the expression of certain biomarkers, such as Ki67, which reflects cell proliferation (4).

Previous studies have indicated that Ki67 is a prognostic indicator for renal tumors, where high expression is associated with aggressive behavior, poor prognosis, and adverse clinicopathological features, potentially serving as a marker for risk stratification and even a therapeutic target in renal tumors (5). For patients with renal tumors under surveillance without surgical indication, Ki67 can serve as a useful predictive marker for identifying suspicious lesions that are highly proliferative. Early detection of such highly proliferative tumors can enhance the precision and effectiveness of treatment, prevent disease progression, and may extend overall survival (6). However, due to the tumor heterogeneity, the Ki67 expression level assessed from the biopsy sample evaluates only a small specimen and may not accurately represent the entire tumor (7). Additionally, biopsy is invasive and cannot dynamically monitor Ki67 expression during tumor follow-up, which limits the application of biopsy techniques in assessing Ki67 expression level.

The high cost and low detection rates of liquid biopsy techniques, such as circulating tumor cells or circulating tumor DNA, have limited their application in detecting Ki67 expression levels in SRMs (8). Imaging techniques, which can directly or indirectly reflect histopathological changes caused by gene and cytokine expression, are currently the most commonly used non-invasive tools for detecting Ki67 expression levels in tumors, but the accuracy of predicting tumor invasiveness or Ki67 expression levels from imaging directly is relatively low (9). Radiomics employs advanced analysis methods to extract numerous features from medical images at high throughput, transforming medical images into mineable high-dimensional data. By analyzing the feature data through automated or semi-automated software, it can provide more quantitative and more reliable information than visual observation (10). Some studies have utilized radiomics technology to predict the Ki67 expression level in renal tumors (11–13). However, these studies failed to provide detailed reproducibility protocols, used only one or a few algorithms to build predictive models, and lacked clinical translation plans, which limited their application. More importantly, these studies focused on renal tumors of all sizes without specific reference to SRMs. SRMs exhibit distinct biological behavior compared to larger renal tumors, and directly applying models developed for all renal tumor sizes to SRMs without assessing their applicability is problematic. For SRMs, where active surveillance is particularly relevant, no specialized non-invasive tool currently exists for accurately predicting Ki67 expression, which is crucial for clinical decision-making.

Therefore, this study focuses on SRMs and employs artificial intelligence algorithms based on clinical characteristics and computed tomography (CT) radiomics to predict the Ki67 expression level. The arterial phase of CT was chosen as the basis for model construction in the study, because Ki67 expression is closely associated with tumor angiogenesis and blood perfusion, which can be effectively captured by arterial phase imaging (14). This non-invasive and convenient tool will enable a more comprehensive and accurate understanding of SRMs, allowing for preoperative assessment of tumor proliferation, risk, invasiveness, benign or malignant tendencies, and prognosis, thereby aiding clinical diagnosis and treatment of SRM patients and promoting personalized treatment strategies.

## 2 Materials and methods

### 2.1 Data collection and processing

This study retrospectively collected data from SRM patients at the First Affiliated Hospital of Guangzhou Medical University (FAHGZMU) and Sun Yat-sen Memorial Hospital (SYSMH) from January 2015 to June 2024. Clinical baseline characteristics, preoperative renal-related CT scans within 30 days, and pathological Ki67 expression were extracted through the hospital information system. Inclusion criteria included: (1) patients underwent surgical resection of renal parenchymal tumors and confirmed to have pathological Ki67 expression levels; (2) renal CT scans conducted within 30 days before surgery, including the arterial phase; (3) the maximum diameter of the renal tumor shown on CT did not exceed 4 centimeters (cm); and (4) complete clinical baseline characteristics were available. Exclusion criteria included: (1) pathology obtained only through renal tumor biopsy; and (2) the arterial phase of CT of poor quality, such as the presence of artifacts or unclear tumor boundaries that are difficult to identify.

Clinical baseline characteristics included age, sex assigned at birth (sex), body mass index (BMI), tumor size, tumor laterality, history of previous or existing other cancers (other cancer), Neutrophil-to-Lymphocyte Ratio (NLR), and estimated Glomerular Filtration Rate (eGFR). Age was categorized into two groups: < 65 years and ≥ 65 years. BMI was calculated as weight in kilograms divided by the square of height in meters and then divided into normal (18.5 ≤ BMI < 25) or abnormal groups. Tumor size was categorized into four groups: 0 < x ≤ 1 cm, 1 < x ≤ 2 cm, 2 < x ≤ 3 cm, and 3 < x ≤ 4 cm. A history of previous or existing other malignant tumors was included in the study because it may indicate a constitutional tendency towards tumorigenesis, which could be associated with tumor aggressiveness and prognosis (15). NLR, which has been shown to correlate with the pathological subtype, grade, stage, and biological aggressiveness of renal tumors (16, 17), was also included in the study. NLR was divided into two groups, < 3 and ≥ 3, as previous studies suggest that categorizing NLR using a cutoff of 3 has more clinical significance (18). The varying aggressiveness of renal tumors may impose different burdens on the kidney, thereby affecting eGFR, which was calculated using the CKD-EPI Creatinine Age, Sex Equation (2021) (19). The formula is: 142 * min(Scr/k,1)^α * max(Scr/k,1)^(-1.200) * 0.9938^age * 1.012[if female], where Scr is serum creatinine, k is 0.7 for females and 0.9 for males, α is -0.241 for females and -0.302 for males, min indicates the minimum of Scr/k or 1, and max indicates the maximum of Scr/k or 1. eGFR was then divided into normal (eGFR ≥ 90) or abnormal groups.

During CT scanning, non-ionic iodinated contrast material was administered at a dose of 1.5 ml/kg (maximum volume 100 ml) through the right antecubital vein using a power injector at a flow rate of 2.5 ml/s. The arterial phase was acquired 25–30 seconds after contrast injection, the venous phase at 60–70 seconds, and the excretory phase at 2–3 minutes. The scan slice thickness was 2 mm, and the reconstruction thickness was 2 mm. Image reconstruction was performed using both soft tissue and sharp kernels. Subsequently, CT images of the patients were imported into the 3D-slicer software (version 5.4.0) in Digital Imaging and Communications in Medicine format, and the arterial phase, which has been confirmed to have research value in previous studies, was selected to extract radiomics features (12). The researchers constructed a predictive model using only the arterial phase of CT to differentiate Ki67 expression levels across renal cell carcinomas of all sizes (12), thereby demonstrating the feasibility of using the arterial phase alone. Additionally, employing only the arterial phase simplifies the workflow, avoids noise and redundancy from other phases, and reduces both model construction and clinical application costs, facilitating efficient use. Moreover, the arterial phase is the most standardized and widely used in renal CT imaging across most centers, which is conducive to model dissemination (20). Although other phases were not considered in the study, it is worth noting that they may provide **Supplementary Information** about the tumor, enriching the feature set and potentially enhancing model accuracy and generalizability.

The region of interest (ROI) for the renal tumor was segmented semi-automatically, initially using the Segment Editor module of the 3D-slicer software to automatically outline, followed by manual adjustment of the ROI on each slice by researcher to ensure that the ROI fit closely to the tumor and did not extend beyond its boundaries. By combining the continuous ROI slices, a three-dimensional volume of interest (VOI) was formed. Radiomics features of the VOI were extracted using the SlicerRadiomics extension plugin, and the images were preprocessed before extraction, including resampling to unify voxel dimensions to 1*1*1 mm^3 to meet isotropy, discretization with a Bin Width of 25 HU to reduce noise and standardize intensity, and application of Gaussian kernel function transformations and wavelet transformations with “1,2,3,4,5” Log kernel sizes and Wavelet-based features to extract features based on LoG filter kernel wavelet transformations (**Supplementary Figure 1**). A total of 1316 radiomics features were extracted, with specific annotations available on the PyRadiomics official website (http://pyradiomics.readthedocs.io/en/latest).

**Figure 1:**
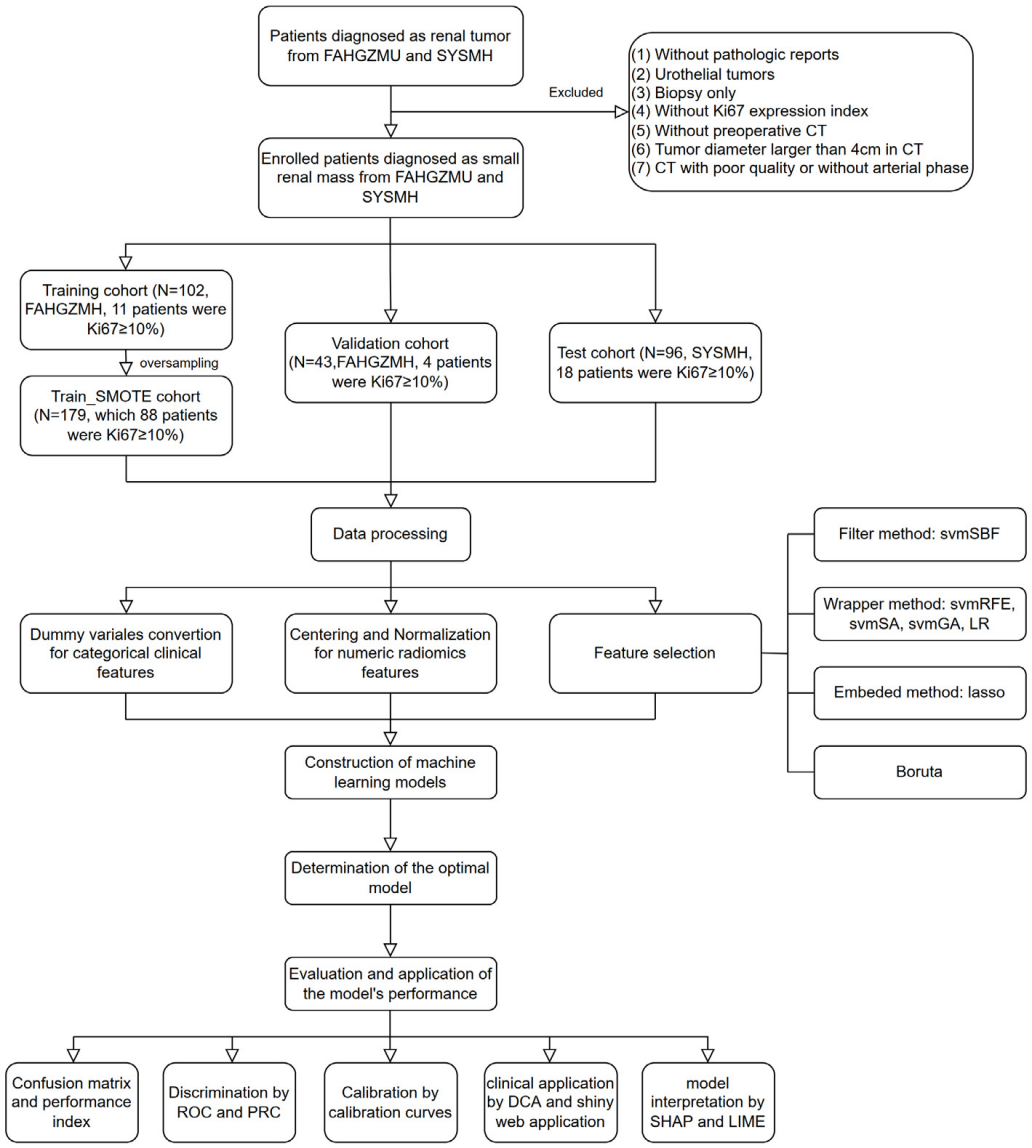
The flowchart of the study. FAHGZMU, First Affiliated Hospital of Guangzhou Medical University; SYSMH, Sun Yat-sen Memorial Hospital; svmSBF, Support Vector Machine-Selection By Filter; svmRFE, Support Vector Machine-Recursive Feature Elimination; svmSA, Support Vector Machine-Simulated Annealing; svmGA, Support Vector Machine-Genetic Algorithms; LR, Logistic Regression; ROC, receiver operator characteristic; PR, precision-recall; DCA, decision curve analysis; SHAP, Shapley Additive exPlanations; LIME, local interpretable model-agnostic explanations.

Ultimately, 148 patients from FAHGZMU and 96 patients from SYSMH were included in the study. Among them, the high Ki67 expression group, defined as Ki67 ≥ 10%, consisted of 25 and 15 cases, respectively. We reviewed studies on the impact of Ki67 expression levels on the biological behavior and prognosis of renal tumors and found a wide range of Ki67 cutoff values used for grouping, with the 10% choice being the most common. Studies that selected 10% as the cutoff included a large number of patients, up to 401, and involved renal tumor histological types that included all stages of clear cell renal cell carcinoma and non-clear cell renal cell carcinoma with follow-up times exceeding 100 months, and endpoints including overall survival, cancer-specific survival and disease-free survival (21, 22). These large, comprehensive cohort studies provide a reference for the choice of Ki67 cutoff values, so we selected 10% as the Ki67 cutoff value, as it may be the most meaningful threshold for prognostic evaluation. Furthermore, patients from FAHGZMU were randomly divided into training and validation cohorts in a ratio of 7:3, while patients from SYSMH served as the test cohort. Considering the class imbalance of Ki67 expression levels, the training cohort was subjected to the Synthetic Minority Over-sampling Technique (SMOTE) algorithm for oversampling to balance the proportion of the high and low Ki67 expression groups (23), forming a dataset with a nearly 1:1 ratio, referred to as the train_SMOTE cohort. The train_SMOTE cohort was used for feature selection and model construction, while the validation and test cohorts were used for model evaluation. All categorical variables were converted into dummy variables in these three cohorts, and continuous variables were standardized using Z-score normalization based on the mean and standard deviation of the train_SMOTE cohort. The study flowchart is detailed in **Figure 1**.

### 2.2 Feature selection and model construction

Images of 30 randomly selected patients were segmented by another researcher, and intraclass correlation coefficient (ICC) was analyzed on the radiomics features extracted by different researchers, among which features with ICC > 0.75 were reserved for subsequent analysis. Correlation analysis was performed on both clinical baseline characteristics and radiomics features to identify and eliminate highly linearly correlated features, which were defined as having Pearson correlation coefficients ≥ 0.9. Subsequently, a variety of dimensionality reduction techniques were employed to refine the feature set: (1) Filter methods: Support Vector Machine-Selection By Filter (svmSBF); (2) Wrapper methods: Support Vector Machine-Recursive Feature Elimination (svmRFE), Support Vector Machine-Simulated Annealing (svmSA), Support Vector Machine-Genetic Algorithms (svmGA), and Logistic Regression (LR); (3) Embedded methods: least absolute shrinkage and selection operator (lasso); (4) Boruta. Since the number of clinical baseline features is relatively small, dimensionality reduction is achieved after applying the above methods. For radiomics features, due to their high dimensionality, an intersection analysis of features selected by different methods was further conducted to obtain the final reduced feature set. The reduced clinical features and radiomics features were then combined to form multiple feature subsets, providing a foundation for subsequent model construction.

A variety of artificial intelligence algorithms were used to build models, including Adaptive Boosting, Discriminant Analysis, eXtreme Gradient Boosting, Gaussian Process, Generalized Linear Model, Logistic Regression, k-Nearest Neighbors, Multivariate Adaptive Regression Spline, Multilayer Perceptron, Neural Network, Naive Bayes, Partial Least Squares, Tree Model, Random Forest, Rotation Forest, Support Vector Machines, Adjacent Categories Probability Model, Multinomial Regression, Ordinal Regression, Radial Basis Function Network, Nearest Shrunken Centroids, Non-Informative Model. During model training, 5-fold cross-validation was implemented, and the hyperparameter tuning strategies built into each algorithm were used to optimize the model performance. By applying these artificial intelligence-based models on the train_SMOTE cohort, validation cohort, and test cohort, predictions could be obtained, and the area under the receiver operating characteristic curve (AUROC) of the models could be calculated accordingly. Ultimately, the models were ranked based on their AUROC on the validation cohort, leading to the selection of the best-performing predictive model.

### 2.3 Model performance evaluation, interpretability, and web application development

The best threshold of the Receiver Operating Characteristic (ROC) curve for the final predictive model was set as the binary classification threshold, with samples above this threshold being classified as high Ki67 expression group, and vice versa. Based on the actual and predicted labels, the confusion matrix could be calculated, where True Positive (TP) represents actual positive and predicted positive, False Positive (FP) represents actual positive and predicted negative, True Negative (TN) represents actual negative and predicted negative, and False Negative (FN) represents actual negative and predicted positive. From this, model performance metrics could be calculated: (1) Precision, equivalent to Positive Predictive Value, calculated as Precision = TP/(TP+FP); (2) Recall, equivalent to Sensitivity and True Positive Rate, calculated as Recall = TP/(TP+FN); (3) Specificity, equivalent to True Negative Rate, calculated as Specificity = TN/(TN+FP); (4) Negative Predictive Value (NPV), calculated as NPV = TN/(TN+FN); (5) Balanced accuracy, which is more meaningful than Accuracy in imbalanced binary classification prediction models, calculated as Balanced accuracy = (Sensitivity+Specificity)/2 = (Recall+Specificity)/2. Additionally, the discriminative power of the final model was assessed using the ROC curve and Precision-Recall (PR) curve, while calibration and clinical usefulness performance were assessed through calibration curves and Decision Curve Analysis (DCA).

Since artificial intelligence algorithms are often considered “black box” models that are difficult to interpret directly, numerous methods have been developed to explain artificial intelligence-based models (24). At both the dataset and individual observation levels, Shapley Additive exPlanations (SHAP) (25) values were calculated, which is a tool for measuring the contribution of variables to model predictions, with positive values indicating an increase in the prediction probability and negative values indicating a decrease. Furthermore, local interpretable model-agnostic explanations (LIME) was used to explain model predictions at the individual observation level, which approximates the decision process of the original model locally by generating virtual samples around a specific observation and then training a simpler, transparent model (26).

To facilitate the clinical translation of the model and to make it easily applicable in actual clinical settings, we developed a Web application using the R package “Shiny”, which integrates the steps of data standardization and model prediction. It consists of two main components: The first part allows users to input model-related predictive factors and obtain prediction results, while the second part provides detailed information about the article and the model, including model performance, interpretation of predictive factors, and relevant information on image processing. Users are required to segment small renal masses on the arterial phase of renal CT using the 3D-Slicer software and extract radiomics features using the settings provided in this study. Upon inputting the raw values of the radiomics features and selecting relevant clinical information, the Web application will generate the predicted Ki67 expression level. This integrated web application aims to enhance the practicality and accessibility of the model, enabling it to support clinical decision-making.

### 2.4 Statistical analysis

All statistical analyses were performed using R version 4.3.3 (https://www.r-project.org/), with statistical significance defined as a two-tailed *P* < 0.05.

For clinical baseline data, which are all categorical variables, the chi-square test was used to calculate expected frequencies; if the frequencies were appropriate for the chi-square test, it was applied. If not, the Fisher’s exact test was utilized for the analysis. The outcomes for categorical variables were depicted as “number (percentage)”.

In subsequent analyses, we utilized 7 methods for the selection of predictive variables: Support Vector Machine-Selection By Filter (svmSBF), Support Vector Machine-Recursive Feature Elimination (svmRFE), Support Vector Machine-Simulated Annealing (svmSA), Support Vector Machine-Genetic Algorithms (svmGA), Logistic Regression (LR), least absolute shrinkage and selection operator (lasso), and Boruta; and constructed predictive models using 22 classes of artificial intelligence algorithms: Adaptive Boosting, Discriminant Analysis, eXtreme Gradient Boosting, Gaussian Process, Generalized Linear Model, Logistic Regression, k-Nearest Neighbors, Multivariate Adaptive Regression Spline, Multilayer Perceptron, Neural Network, Naive Bayes, Partial Least Squares, Tree Model, Random Forest, Rotation Forest, Support Vector Machines, Adjacent Categories Probability Model, Multinomial Regression, Ordinal Regression, Radial Basis Function Network, Nearest Shrunken Centroids, Non-Informative Model.

## 3 Results

### 3.1 Baseline characteristics

After applying the inclusion and exclusion criteria, the study ultimately included 145 patients from FAHGZMU and 96 patients from SYSMH. Among the patients in this center, there were 33 cases (13.69%) and 18 cases (18.75%) in the high Ki67 expression group, respectively. Additionally, the cases from both centers showed a similar distribution in other clinical baseline characteristics, with no statistically significant differences. Specifically, SRM patients included in the study were mainly males under 65 years old, without a history of previous or existing other cancers, with tumor diameters mostly over 2 centimeters, slightly more common on the right side, and with NLR values mostly less than 3. The proportions of normal and abnormal for BMI and eGFR were roughly equal (**Table 1**).

**Table 1:** Baseline characteristics of small renal mass patients.

	FAHGZMU			SYSMH	*P* for FAHGZMU and SYSMH
	All, N=145	Train, N=102	Val, N=43	Test, N=96	
Age (y)					0.903
<65	181 (75.10%)	78 (76.47%)	30 (69.77%)	73 (76.04%)	
≥65	60 (24.90%)	24 (23.53%)	13 (30.23%)	23 (23.96%)	
Sex					0.267
Female	97 (40.25%)	48 (47.06%)	15 (34.88%)	34 (35.42%)	
Male	144 (59.75%)	54 (52.94%)	28 (65.12%)	62 (64.58%)	
BMI					0.694
Normal	123 (51.04%)	56 (54.90%)	20 (46.51%)	47 (48.96%)	
Abnormal	118 (48.96%)	46 (45.10%)	23 (53.49%)	49 (51.04%)	
Other cancer					0.239
Yes	20 (8.30%)	9 (8.82%)	6 (13.95%)	5 (5.21%)	
No	221 (91.70%)	93 (91.18%)	37 (86.05%)	91 (94.79%)	
Diameter (cm)					0.606
0<x ≤ 1	7 (2.90%)	2 (1.96%)	2 (4.65%)	3 (3.12%)	
1<x ≤ 2	49 (20.33%)	24 (23.53%)	9 (20.93%)	16 (16.67%)	
2<x ≤ 3	85 (35.27%)	39 (38.24%)	13 (30.23%)	33 (34.38%)	
3<x ≤ 4	100 (41.49%)	37 (36.27%)	19 (44.19%)	44 (45.83%)	
Laterality					0.473
Left	111 (46.06%)	47 (46.08%)	23 (53.49%)	41 (42.71%)	
Right	130 (53.94%)	55 (53.92%)	20 (46.51%)	55 (57.29%)	
NLR					0.115
<3	180 (74.69%)	80 (78.43%)	34 (79.07%)	66 (68.75%)	
≥3	61 (25.31%)	22 (21.57%)	9 (20.93%)	30 (31.25%)	
eGFR					0.692
Normal	123 (51.04%)	52 (50.98%)	20 (46.51%)	51 (53.12%)	
Abnormal	118 (48.96%)	50 (49.02%)	23 (53.49%)	45 (46.88%)	
Ki67 expression					0.096
<10%	208 (86.31%)	91 (89.22%)	39 (90.70%)	78 (81.25%)	
≥10%	33 (13.69%)	11 (10.78%)	4 (9.30%)	18 (18.75%)	

FAHGZMU, First Affiliated Hospital of Guangzhou Medical University; SYSMH, Sun Yat-sen Memorial Hospital; Train, training cohort; Val, validation cohort; Test, test cohort; BMI, body mass index; Other cancer, history of previous or existing other cancers; NLR, Neutrophil-to-Lymphocyte Ratio; eGFR, estimated Glomerular Filtration Rate; Normal of BMI and eGFR represents 18.5 ≤ BMI < 25 and eGFR ≥ 90, respectively. Calculation formulas: BMI = weight/(height)^2; NLR = Blood neutrophils/Blood lymphocytes; eGFR = 142 * min(Scr/k,1)^α * max(Scr/k,1)^(-1.200) * 0.9938^age * 1.012[if female], where Scr is serum creatinine, k is 0.7 for females and 0.9 for males, α is -0.241 for females and -0.302 for males, min indicates the minimum of Scr/k or 1, and max indicates the maximum of Scr/k or 1.

Subsequently, the FAHGZMU cohort was randomly split into a training cohort and a validation cohort in a ratio of 7:3, with the training cohort including 11 cases in the high Ki67 expression group, accounting for 10.78%. To address the issue of class imbalance, we employed the SMOTE oversampling strategy, increasing the number of samples in the high Ki67 expression group from 11 to 88. After oversampling, the distribution of baseline characteristics in the high Ki67 expression group remained consistent with the pre-oversampling distribution, showing no statistically significant differences (**Supplementary Table 1**). This was then combined with the original 91 cases in the low Ki67 expression group to form the train_SMOTE cohort, which was nearly a 1:1 ratio and used for subsequent model construction.

In previous studies on predicting Ki67 levels for other tumors, most investigations utilized single-center data for model construction and validation. Only a few studies, like ours, incorporated external data to further test the models, such as those in lung cancer (27) and gastrointestinal stromal tumors (28). Additionally, it is worth mentioning that a few studies, due to imbalanced outcome classes, also employed SMOTE for oversampling (29, 30).

### 3.2 Feature selection and model construction

**Figure 2:**
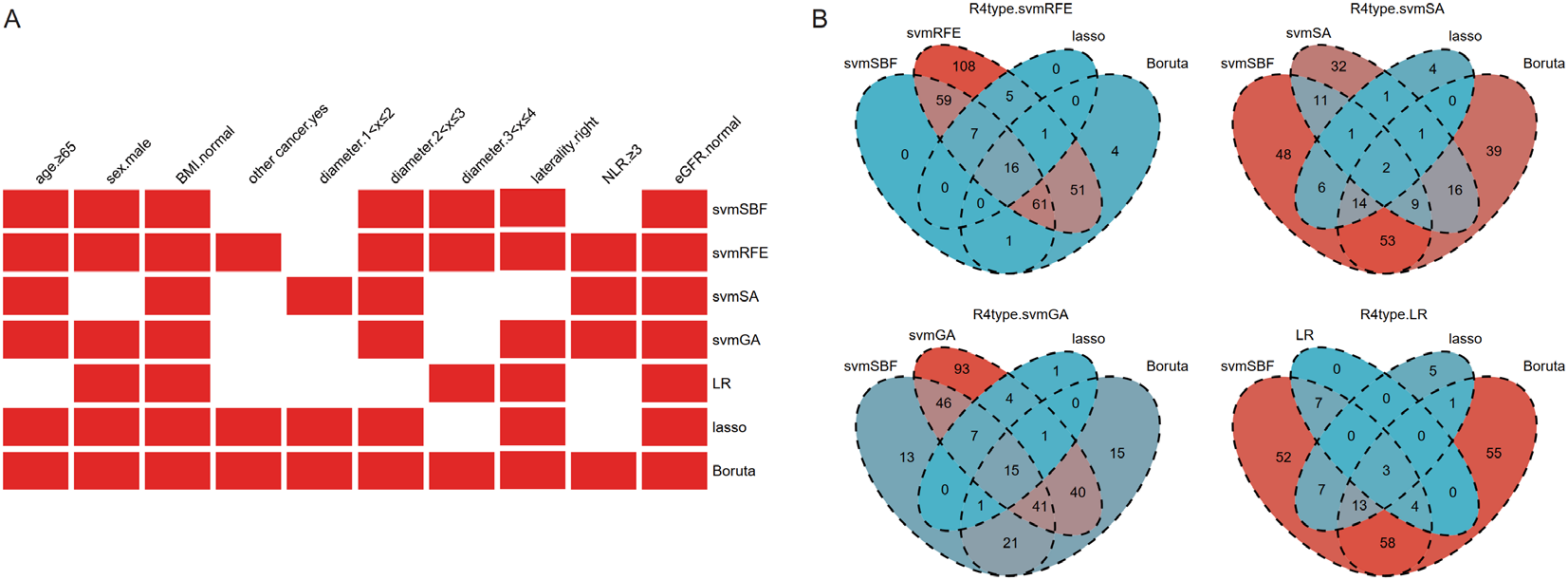
The results of feature selection of clinical baseline characteristics **(A)** and radiomics features **(B)**. Given that the number of radiomics features after selection was still large, intersection was further used to refine the feature set. BMI, body mass index; other cancer, history of previous or existing other cancers; NLR, Neutrophil-to-Lymphocyte Ratio; eGFR, estimated Glomerular Filtration Rate; svmSBF, Support Vector Machine-Selection By Filter; svmRFE, Support Vector Machine-Recursive Feature Elimination; svmSA, Support Vector Machine-Simulated Annealing; svmGA, Support Vector Machine-Genetic Algorithms; LR, Logistic Regression; R4type, three methods (svmSBF, lasso, Boruta) together intersected with the wrapper methods (svmRFE, svmSA, svmGA, and LR in sequence).

Feature selection was performed separately on clinical baseline characteristics and radiomics features. There was no high correlation among the clinical baseline characteristics, so all were used for feature selection. Through different selection methods, seven optimal subsets were ultimately produced, with one subset including all 10 clinical baseline characteristics (**Figure 2A**).

The 1316 radiomics features extracted by 3D-slicer were reduced to 337 after ICC analysis and removal of highly linear correlation features. Various dimensionality reduction methods were applied, with svmSBF, svmRFE, svmSA, svmGA, LR, lasso, and Boruta selecting 144, 308, 73, 247, 14, 29, and 134 features, respectively. Given that the number of features after selection was still large, we further refined the feature set through intersection. The filter method svmSBF, the embedded methods lasso, and Boruta, these three methods were collectively intersected with the wrapper methods svmRFE, svmSA, svmGA, and LR in sequence. Ultimately, this process respectively identified 16, 2, 15, and 3 radiomics features (**Figure 2B**), forming four optimal subsets of radiomics features.

**Figure 3:**
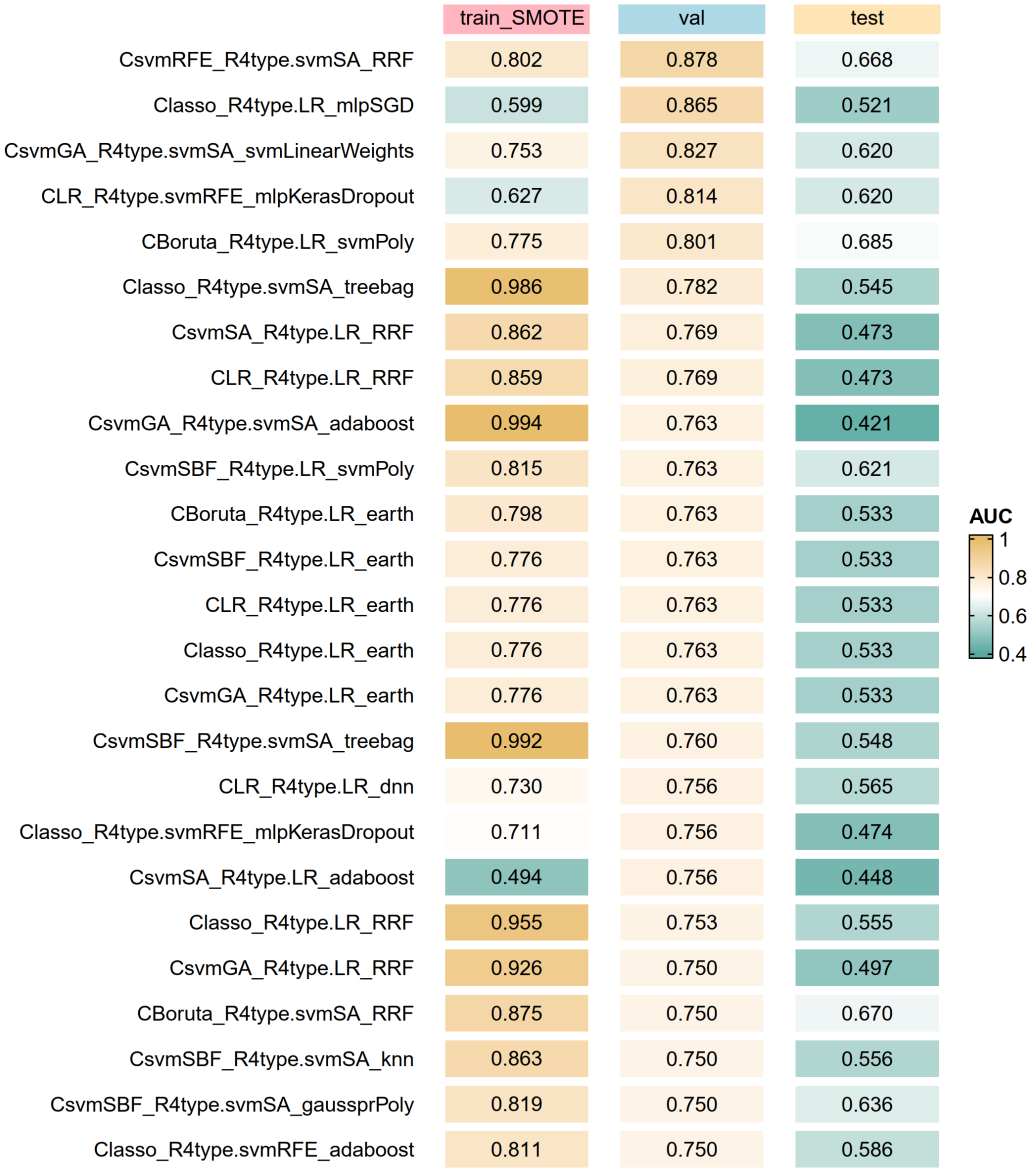
Top artificial intelligence algorithms for model optimization according to the AUROC of the validation cohort. The algorithm label (C_R_M) represents clinical features (C) and radiomics features (R) are used together to build the model (M). train_SMOTE, train_SMOTE cohort; val, validation cohort; test, test cohort; AUROC, the area under the receiver operating characteristic curve.

The optimal subsets of clinical baseline characteristics and radiomics features were combined one by one, resulting in 28 datasets used for constructing artificial intelligence-based models. Using the R package caret, we trained 66 models of 22 types of algorithms on the train_SMOTE cohort. The specific names of these algorithms and corresponding parameters of method in the caret package are shown in **Supplementary Table 2**. The AUROC of each model in the train_SMOTE cohort, validation cohort, and test cohort was calculated and ranked based on the values from the validation cohort. Some model results were visualized through heatmaps (**Figure 3**), while the detailed results of all models are presented in **Supplementary Table 3**. Among all models, the best performing model was CsvmRFE_R4type.svmSA_RRF, indicating that its clinical features (C) were selected using the svmRFE method, while its radiomics features (R), LoG(σ=2)_firstorder_Maximum and LoG(σ=2)_GLSZM_LowGrayLevelZoneEmphasis, were determined through the intersection analysis of svmSA with the other three methods, and it is a regularized random forest (RRF) model constructed based on these variables. This optimal model demonstrated AUROC values of 0.802, 0.878, and 0.668 in the train_SMOTE cohort, validation cohort, and test cohort, respectively.

We employed a variety of variable selection and model construction methods in the study, which is rare in existing studies on predicting Ki67 levels in renal tumors or other tumors. While a few studies might have used multiple variable selection methods, such as in pituitary adenomas (31), or multiple model construction methods, such as the five machine learning algorithms used in breast cancer (32) and medulloblastoma studies (33), the majority of studies selected only one or a few methods. This may lead to the neglect of other potential approaches.

### 3.3 Model performance

**Figure 4:**
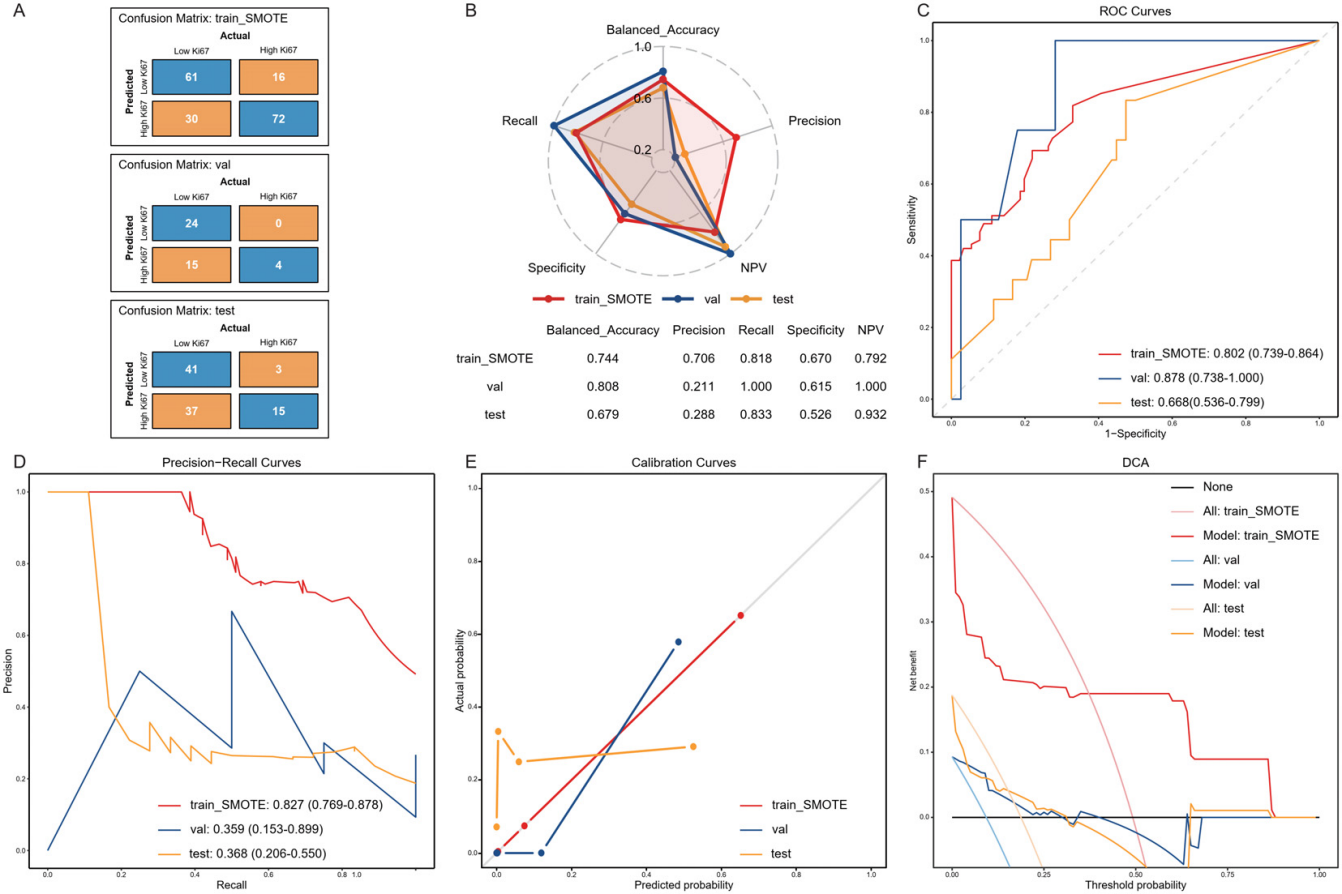
Model Performance. Confusion matrices **(A)**, performance metrics **(B)**, ROC curves **(C)**, PR curves **(D)**, calibration curves **(E)**, and DCA **(F)** of the final model in the train_SMOTE, validation and test cohort. The results of ROC and PR curves was presented in the format of “the area under the curve (95% confidence interval)”. train_SMOTE, train_SMOTE cohort; val, validation cohort; test, test cohort; NPV, Negative Predictive Value; ROC, receiver operating characteristic; DCA, Decision Curve Analysis.

Based on the best threshold of 0.003 determined by the ROC curve of the optimal model in the train_SMOTE cohort, the predicted values were transformed into binary classification results. Consequently, the confusion matrices (**Figure 4A**) and performance metrics (**Figure 4B**) were calculated. The balanced accuracy of the model in the train_SMOTE cohort, validation cohort, and test cohort was 0.744, 0.808, and 0.679, respectively. Regarding other performance indices, the precision, recall, specificity, and NPV of the training cohort were 0.706, 0.818, 0.670, and 0.792, respectively. In the validation and test cohorts, the recall (1.000 and 0.833) and NPV (1.000 and 0.932) increased, while specificity slightly decreased (0.615 and 0.526), and precision decreased more significantly (0.211 and 0.288). The high recall and NPV, along with the low precision, suggest that the model could effectively identify most high Ki67 expression cases but also produced relatively high false positives.

The model’s discrimination was demonstrated through ROC and PR curves. The AUROC results were consistent with those shown in the model optimization, with values of 0.802, 0.878, and 0.668 in the train_SMOTE cohort, validation cohort, and test cohort, respectively (**Figure 4C**). The possible reasons for the lower AUROC value in the test cohort may include: (1) the small sample size of the internal datasets, which may limit the model’s generalizability; (2) oversampling of the minority class with high Ki67 expression during model training, which may reduce model performance in real-world cohorts; and (3) the exclusive use of radiomics features from arterial phase CT in model construction, which may lead to the neglect of potential predictive factors in other imaging modalities and phases. The area under the PR curve (AUPRC) was significantly reduced in the validation cohort and test cohort, with values of 0.359 and 0.368, respectively, compared to 0.827 in the train_SMOTE cohort (**Figure 4D**). Although the ratio of outcome variable in the train_SMOTE cohort was close to 1:1, class imbalance still existed in the validation and test cohort, with the high Ki67 expression group accounting for 9.30% and 18.75%, respectively, which may be the main reason for the lower AUPRC (34). In addition, we also plotted calibration curves (**Figure 4E**) and DCA (**Figure 4F**) to demonstrate the model’s calibration and clinical usefulness.

Compared with other studies on predicting Ki67 levels, most investigations typically only had internal datasets, making it impossible to assess their external generalizability. In these studies, the AUC values for their training and validation cohorts were usually around 0.8, similar to our internal dataset. A few studies had poorer discrimination performance, such as an AUC of 0.69 (33), or better performance, with AUC values of 0.95 and 0.86 in the training and validation cohorts, respectively (32). Among the few studies involving external datasets, their external cohort performance was slightly worse than the internal data but generally reached above 0.7 (27, 28), showing slightly better generalizability than our study.

### 3.4 Model interpretability

We attempted to explain the “black box” of the artificial intelligence-based model, with variable importance and SHAP values used to reveal the contribution of each variable to the model’s predictions. The results showed that the radiomics feature LoG(σ=2)_firstorder_Maximum had a significant contribution to the model; however, the contributions of other included variables could not be quantified through these two methods (**Supplementary Figures 2A-C**). Consistent results were obtained through local analysis of specific observations (**Supplementary Figures 2D-E**).

To clarify whether other predictive variables do not play a role in the model’s predictions, we attempted to predict Ki67 expression levels using only the feature LoG(σ=2)_firstorder_Maximum. The binary classification of the prediction results was based on the best threshold of ROC curve of this feature in the train_SMOTE cohort. The results showed that when relying solely on LoG(σ=2)_firstorder_Maximum for prediction, the model’s AUROC and balanced accuracy significantly decreased compared to before, and other performance metrics also decreased to varying degrees (**Supplementary Figure 2F**). This finding suggests that the model’s overall performance was compromised after the removal of other variables, thus confirming that other variables also play within the model, although variable importance and SHAP did not adequately explain or quantify their impact.

Furthermore, we used LIME to explain the optimal model, which is an algorithm that constructs a more understandable, lasso, to explain the prediction results for specific observations. The analysis results revealed the importance of variables that significantly influenced the prediction results for specific observations, showing that LoG(σ=2)_firstorder_Maximum was indeed the most important predictive variable, while other variables were not without effect (**Supplementary Figure 2G**).

Nomograms can illustrate the decision-making process of logistic regression by quantifying the risk scores of predictive factors. In contrast, machine learning models usually require indirect methods for interpretation. We used SHAP and LIME to elucidate the mechanisms of our models to some extent. This is an improvement compared to previous machine learning models for predicting tumor Ki67 levels, which did not attempt to explain the models (29, 31).

### 3.5 Web application development

We built an interactive web application where users can input the values of relevant predictive variables to output the predicted Ki67 level grouping results (https://doctorlinjy.shinyapps.io/smallrenalmasski67prediction/).

Models based on logistic regression can be enhanced in their applicability through nomograms. However, previous models based on machine learning algorithms, such as the k-Nearest Neighbors model for pituitary adenomas (31) and the random forest model for gliomas (29), typically did not provide a way to use the model. This limited their clinical translation. In contrast, the web application we developed facilitates the use of the established models by users.

## 4 Discussion

Advances and widespread adoption of imaging technologies have led to a significant increase in the number of incidentally detected renal masses, particularly SRMs with a diameter not exceeding 4 centimeters (35). Although the prognosis for the majority of SRMs is relatively favorable, a subset of them still exhibit robust proliferation and invasiveness (3). Analyzing the cell proliferation marker Ki67 allows for the assessment of tumor growth and invasiveness, which is crucial for predicting the aggressiveness of SRMs, risk stratification, and clinical decision-making (4, 5). Radiomics techniques have the potential to enhance the accuracy of tumor diagnosis and have shown promise in evaluating tumor characteristics, assisting differential diagnosis, and optimizing clinical decisions (36). Numerous studies have demonstrated the effectiveness of radiomics techniques in preoperatively predicting Ki67 expression levels in various tumors. In the field of renal tumor, Scrima and colleagues found a significant statistical correlation between CT texture features and Ki67 expression in SRMs, but their study did not delve further (11); Yang and colleagues constructed an xgboost-based predictive model based on arterial phase radiomics features, however, their study included renal tumors of all sizes, not specifically focusing on SRMs (12).

This study collected data from SRM patients at two centers and grouped them based on Ki67 expression, among which patients with Ki67 ≥10% were classified as high expression group. Clinical baseline characteristics of the patients were collected, and tumor segmentation of the arterial phase CT images was performed using 3D-slicer software, extracting 1316 radiomics features. By reducing the dimensionality of clinical and radiomics features, a regularized random forest model was ultimately constructed to predict Ki67 expression levels. The model demonstrated AUROC values of 0.802, 0.878, and 0.668, and balanced accuracy of 0.744, 0.808, and 0.679 in the training, validation, and test cohorts, respectively. Additionally, calibration curves and DCA showed that the model has a certain degree of calibration and clinical usefulness value. To facilitate the use of the model, we also developed a web application.

The artificial intelligence-based predictive model allows for non-invasive preoperative assessment of Ki67 expression in SRM patients, enabling preliminary judgment of the tumor’s proliferative capacity, risk, invasiveness, and malignant potential and prognosis, increasing understanding of its natural course. This aids in providing more diagnostic and therapeutic evidence for SRM patients, assisting clinical decision-making, and promoting personalized precision therapy. For SRM patients predicted to have low Ki67 expression, there will be a basis for considering delay treatment and potentially performing renal tumor biopsy before deciding on subsequent treatment; whereas for patients predicted to have high Ki67 expression, there is a cautionary effect, indicating that preoperative treatment plans may be considered, or higher requirements may be necessary to achieve the standard of negative surgical margin.

The model interpretability highlights the crucial role of the radiomics feature in the prediction process. Although other features did not reveal their specific contributions through SHAP analysis, they still possess potential relevance that cannot be ignored. For example, age, gender, and BMI may reflect different physiological and metabolic states of the body, which could potentially be associated with the aggressiveness of renal tumors through their impacts on the molecular level, immune function, and lifestyle (37). Additionally, a history of previous or existing other malignant tumors may indicate a predisposition to tumor development, thereby affecting the aggressiveness and prognosis of renal tumors (15). The NLR has been shown to be associated with the pathological subtype, grade, stage, and biological aggressiveness of renal tumors, which may be reflected through Ki67 expression (16, 17). Furthermore, eGFR, as an indicator of renal filtration function, may vary with different levels of tumor aggressiveness, as more aggressive renal tumors may impose greater burdens on kidney function, resulting in different eGFR levels (19).

Ki67 exhibits significant heterogeneity in renal cell carcinoma, which has important implications for clinical outcomes. Specifically, high Ki67 expression is typically associated with aggressive pathological features, such as tumor necrosis, high-grade nuclear grading, and perirenal fat invasion, and is also linked to increased recurrence risk and significantly reduced survival rates (5). In the studies related to Ki67, the choice of cutoff values varies greatly among different tumors, and even for the same tumor, a unified standard is lacking. This directly affects the assessment of patient prognosis and treatment decision-making. For example, a lower cutoff value can increase sensitivity but carries the risk of overdiagnosis, while a higher cutoff value has the opposite effect. Moreover, the use of different cutoff values in various studies may place the same patient in different risk groups, leading to conflicting prognostic interpretations and thereby obscuring the true value of Ki67 as a prognostic marker. The heterogeneity of Ki67 and the variability in cutoff values undermine its reliability as an independent prognostic tool for RCC, resulting in inconsistent clinical outcomes and suboptimal treatment decisions. Future research needs to establish more standardized detection and evaluation methods, focusing on standardizing the cutoff values for Ki67, and further exploring its application effects in different subtypes or sizes, in order to fully realize the potential of Ki67 in precision oncology.

This study has some limitations. First, the number of cases included in this study is relatively small. Although we collected cases from two centers, only 145 and 96 SRM patients were included, with 33 and 18 cases in the high Ki67 expression group, respectively. Limited by the sample size, we could not perform matching but instead used an oversampling strategy. Although many studies have used oversampling strategies, and the datasets before and after oversampling in our study had similar distributions, whether the data generated by oversampling can accurately represent real-world situations remains to be verified. Second, this study only selected the arterial phase for analysis, which, despite being the most commonly used enhanced phase and having been confirmed in previous studies to have certain performance in predicting Ki67 in renal tumors, did not consider other phases or imaging methods, which may lead to the omission of some useful features that we did not focus on. Third, we selected the best-performing model based on AUROC, which has good performance, but the artificial intelligence-based model is a black box, and variable importance and SHAP cannot explain it well. Although we clarified the input and output processes of the model, its decision-making process is not transparent enough. Lastly, the performance of the constructed model is not satisfactory. In the future, strategies to improve model generalizability are needed. These include increasing the sample size, incorporating data from more centers, ensuring class balance in the original data, including more clinical features, utilizing a wider range of imaging modalities and phases, and developing more transparent artificial intelligence-based models.

## 5 Conclusions

This study developed an artificial intelligence-based predictive model for non-invasively assessing the Ki67 expression level in patients with small renal mass, providing valuable reference for clinical decision-making in these patients. However, the model was established based on retrospective analysis of arterial phase CT and clinical characteristics from two centers and still requires validation in prospective cohorts. In the future, it needs to be improved using larger and more diverse datasets, additional imaging modalities and phases, and more advanced radiomics and artificial intelligence methods.

## Data Availability

The original contributions presented in the study are included in the article/**Supplementary Material**. Further inquiries can be directed to the corresponding author.

## Supplementary material

Supplementary Figure 1 Demonstration of the settings for radiomics feature extraction using the SlicerRadiomics extension plugin in the 3D-slicer software.

Supplementary Figure 2 Model Interpretability. Global explanations include variable importance **(A)** and SHAP values **(B, C)**, and local explanations include SHAP values **(D, E)** and LIME **(G)**. Since variable importance and SHAP only show the importance of the radiomics feature LoG(σ=2)_firstorder_Maximum, only the feature was used for prediction, and the results show that the performance metrics drop significantly compared with before **(F)**. BMI, body mass index; other cancer, history of previous or existing other cancers; NLR, Neutrophil-to-Lymphocyte Ratio; eGFR, estimated Glomerular Filtration Rate; train_SMOTE, train_SMOTE cohort; val, validation cohort; test, test cohort; AUROC, the area under the receiver operating characteristic curve; NPV, Negative Predictive Value; SHAP, Shapley Additive exPlanations; LIME, local interpretable model-agnostic explanations.

## References

[1] Finelli A Ismaila N Bro B Durack J Eggener S Evans A. Management of small renal masses: american society of clinical oncology clinical practice guideline. J Clin Oncol. (2017) 35:668–80. doi: 10.1200/jco.2016.69.9645 28095147

[2] Umbreit EC Shimko MS Childs MA Lohse CM Cheville JC Leibovich BC. Metastatic potential of a renal mass according to original tumour size at presentation. BJU Int. (2012) 109:190–4; discussion 4. doi: 10.1111/j.1464-410X.2011.10184.x 21557795

[3] Zhang L Yao L Li X Jewett MA He Z Zhou L. Natural history of renal cell carcinoma: An immunohistochemical analysis of growth rate in patients with delayed treatment. J Formosan Med Assoc = Taiwan yi zhi. (2016) 115:463–9. doi: 10.1016/j.jfma.2015.05.003 26058870

[4] Oda T Takahashi A Miyao N Yanase M Masumori N Itoh N. Cell proliferation, apoptosis, angiogenesis and growth rate of incidentally found renal cell carcinoma. Int J Urol. (2003) 10:13–8. doi: 10.1046/j.1442-2042.2003.00558.x 12534920

[5] Xie Y Chen L Ma X Li H Gu L Gao Y. Prognostic and clinicopathological role of high Ki-67 expression in patients with renal cell carcinoma: a systematic review and meta-analysis. Sci Rep. (2017) 7:44281. doi: 10.1038/srep44281 28287186 PMC5347162

[6] Huang Z Lyu M Ai Z Chen Y Liang Y Xiang Z. Pre-operative prediction of ki-67 expression in various histological subtypes of lung adenocarcinoma based on CT radiomic features. Front Surg. (2021) 8:736737. doi: 10.3389/fsurg.2021.736737 34733879 PMC8558627

[7] Boros M Moncea D Moldovan C Podoleanu C Georgescu R Stolnicu S. Intratumoral heterogeneity for ki-67 index in invasive breast carcinoma: A study on 131 consecutive cases. Appl Immunohistochem Mol Morphol: AIMM. (2017) 25:338–40. doi: 10.1097/pai.0000000000000315 26766125

[8] Crocetto F Falcone A Mirto BF Sicignano E Pagano G Dinacci F. Unlocking precision medicine: liquid biopsy advancements in renal cancer detection and monitoring. Int J Mol Sci. (2024) 25:3867. doi: 10.3390/ijms25073867 PMC11011885 38612677

[9] Yao W Liao Y Li X Zhang F Zhang H Hu B. Noninvasive method for predicting the expression of ki67 and prognosis in non-small-cell lung cancer patients: radiomics. J Healthcare Eng. (2022) 2022:7761589. doi: 10.1155/2022/7761589 PMC8942651 35340222

[10] Lambin P Rios-Velazquez E Leijenaar R Carvalho S van Stiphout RG Granton P. Radiomics: extracting more information from medical images using advanced feature analysis. Eur J Cancer (Oxford England: 1990). (2012) 48:441–6. doi: 10.1016/j.ejca.2011.11.036 PMC4533986 22257792

[11] Scrima AT Lubner MG Abel EJ Havighurst TC Shapiro DD Huang W. Texture analysis of small renal cell carcinomas at MDCT for predicting relevant histologic and protein biomarkers. Abdominal Radiol (New York). (2019) 44:1999–2008. doi: 10.1007/s00261-018-1649-2 29804215

[12] Yang H Lin J Liu H Yao J Lin Q Wang J. Automatic analysis framework based on 3D-CT multi-scale features for accurate prediction of Ki67 expression levels in substantial renal cell carcinoma. Insights Into Imaging. (2023) 14:130. doi: 10.1186/s13244-023-01465-y 37466878 PMC10356689

[13] Yang H Liu H Lin J Xiao H Guo Y Mei H. An automatic texture feature analysis framework of renal tumor: surgical, pathological, and molecular evaluation based on multi-phase abdominal CT. Eur Radiol. (2024) 34:355–66. doi: 10.1007/s00330-023-10016-4 37528301

[14] Fu Q Liu SL Hao DP Hu YB Liu XJ Zhang Z. CT radiomics model for predicting the ki-67 index of lung cancer: an exploratory study. Front Oncol. (2021) 11:743490. doi: 10.3389/fonc.2021.743490 34707991 PMC8542688

[15] Zhou H Huang Y Qiu Z Zhao H Fang W Yang Y. Impact of prior cancer history on the overall survival of patients newly diagnosed with cancer: A pan-cancer analysis of the SEER database. Int J Cancer. (2018) 143:1569–77. doi: 10.1002/ijc.31543 29667174

[16] Viers BR Thompson RH Lohse CM Cheville JC Leibovich BC Boorjian SA. Pre-treatment neutrophil-to-lymphocyte ratio predicts tumor pathology in newly diagnosed renal tumors. World J Urol. (2016) 34:1693–9. doi: 10.1007/s00345-016-1821-7 27052014

[17] Liu H Tang K Chen Z Li Z Meng X Xia D. Comparison and development of preoperative systemic inflammation markers-based models for the prediction of unfavorable pathology in newly diagnosed clinical T1 renal cell carcinoma. Pathol Res Pract. (2021) 225:153563. doi: 10.1016/j.prp.2021.153563 34371466

[18] Boissier R Campagna J Branger N Karsenty G Lechevallier E. The prognostic value of the neutrophil-lymphocyte ratio in renal oncology: A review. Urol Oncol. (2017) 35:135–41. doi: 10.1016/j.urolonc.2017.01.016 28233671

[19] Inker LA Eneanya ND Coresh J Tighiouart H Wang D Sang Y. New creatinine- and cystatin C-based equations to estimate GFR without race. New Engl J Med. (2021) 385:1737–49. doi: 10.1056/NEJMoa2102953 PMC8822996 34554658

[20] Tang Y Gao R Lee HH Xu Z Savoie BV Bao S. Renal cortex, medulla and pelvicaliceal system segmentation on arterial phase CT images with random patch-based networks. Proc SPIE–the Int Soc Optical Eng. (2021) 11596:115961D. doi: 10.1117/12.2581101 PMC8442958 34531632

[21] Gayed BA Youssef RF Bagrodia A Darwish OM Kapur P Sagalowsky A. Ki67 is an independent predictor of oncological outcomes in patients with localized clear-cell renal cell carcinoma. BJU Int. (2014) 113:668–73. doi: 10.1111/bju.12263 23937277

[22] Pinto AE Monteiro P Silva G Ayres JV Soares J. Prognostic biomarkers in renal cell carcinoma: relevance of DNA ploidy in predicting disease-related survival. Int J Biol Markers. (2005) 20:249–56. doi: 10.1177/172460080502000408 16398407

[23] Chawla NV Bowyer KW Hall LO Kegelmeyer WP. SMOTE: synthetic minority over-sampling technique. J Artif Intell Res. (2011) 16:321–57. doi: 10.48550/arXiv.1106.1813. arXiv:1106.813 p.

[24] Karim MR Islam T Shajalal M Beyan O Lange C Cochez M. Explainable AI for bioinformatics: methods, tools and applications. Briefings Bioinf. (2023) 24:bbad236. doi: 10.1093/bib/bbad236 37478371

[25] Nohara Y Matsumoto K Soejima H Nakashima N. Explanation of machine learning models using shapley additive explanation and application for real data in hospital. Comput Methods Programs Biomed. (2022) 214:106584. doi: 10.1016/j.cmpb.2021.106584 34942412

[26] Ali S Akhlaq F Imran AS Kastrati Z Daudpota SM Moosa M. The enlightening role of explainable artificial intelligence in medical & healthcare domains: A systematic literature review. Comput Biol Med. (2023) 166:107555. doi: 10.1016/j.compbiomed.2023.107555 37806061

[27] Yan J Xue X Gao C Guo Y Wu L Zhou C. Predicting the Ki-67 proliferation index in pulmonary adenocarcinoma patients presenting with subsolid nodules: construction of a nomogram based on CT images. Quantitative Imaging Med Surg. (2022) 12:642–52. doi: 10.21037/qims-20-1385 PMC8666773 34993108

[28] Zhang QW Gao YJ Zhang RY Zhou XX Chen SL Zhang Y. Personalized CT-based radiomics nomogram preoperative predicting Ki-67 expression in gastrointestinal stromal tumors: a multicenter development and validation cohort. Clin Trans Med. (2020) 9:12. doi: 10.1186/s40169-020-0263-4 PMC6994569 32006200

[29] Gao M Huang S Pan X Liao X Yang R Liu J. Machine learning-based radiomics predicting tumor grades and expression of multiple pathologic biomarkers in gliomas. Front Oncol. (2020) 10:1676. doi: 10.3389/fonc.2020.01676 33014836 PMC7516282

[30] Li J Liu S Qin Y Zhang Y Wang N Liu H. High-order radiomics features based on T2 FLAIR MRI predict multiple glioma immunohistochemical features: A more precise and personalized gliomas management. PloS One. (2020) 15:e0227703. doi: 10.1371/journal.pone.0227703 31968004 PMC6975558

[31] Ugga L Cuocolo R Solari D Guadagno E D’Amico A Somma T. Prediction of high proliferative index in pituitary macroadenomas using MRI-based radiomics and machine learning. Neuroradiology. (2019) 61:1365–73. doi: 10.1007/s00234-019-02266-1 31375883

[32] Wu L Zhao Y Lin P Qin H Liu Y Wan D. Preoperative ultrasound radiomics analysis for expression of multiple molecular biomarkers in mass type of breast ductal carcinoma in *situ* . BMC Med Imaging. (2021) 21:84. doi: 10.1186/s12880-021-00610-7 34001017 PMC8130392

[33] Zhou L Peng H Ji Q Li B Pan L Chen F. Radiomic signatures based on multiparametric MR images for predicting Ki-67 index expression in medulloblastoma. Ann Trans Med. (2021) 9:1665. doi: 10.21037/atm-21-5348 PMC8667089 34988174

[34] Lv K Cui C Fan R Zha X Wang P Zhang J. Detection of diabetic patients in people with normal fasting glucose using machine learning. BMC Med. (2023) 21:342. doi: 10.1186/s12916-023-03045-9 37674168 PMC10483877

[35] Jaggi A Mastrodicasa D Charville GW Jeffrey RB Jr. Napel S Patel B. Quantitative image features from radiomic biopsy differentiate oncocytoma from chromophobe renal cell carcinoma. J Med Imaging (Bellingham Wash). (2021) 8:54501. doi: 10.1117/1.Jmi.8.5.054501 PMC8423237 34514033

[36] Guo J Liu Z Shen C Li Z Yan F Tian J. MR-based radiomics signature in differentiating ocular adnexal lymphoma from idiopathic orbital inflammation. Eur Radiol. (2018) 28:3872–81. doi: 10.1007/s00330-018-5381-7 29632999

[37] Hötker AM Karlo CA Di Paolo PL Zheng J Moskowitz CS Russo P. Renal cell carcinoma: Associations between tumor imaging features and epidemiological risk factors. Eur J Radiol. (2020) 129:109096. doi: 10.1016/j.ejrad.2020.109096 32559590 PMC8423027

