# Supplementary figures and images for: Combining clinical characteristics with CT radiomics to predict Ki67 expression level of small renal mass based on artificial intelligence algorithms
Source: Front Oncol. 2025 Feb 21;15:1541143. doi: 10.3389/fonc.2025.1541143 (PMC11885116; doi:10.3389/fonc.2025.1541143)

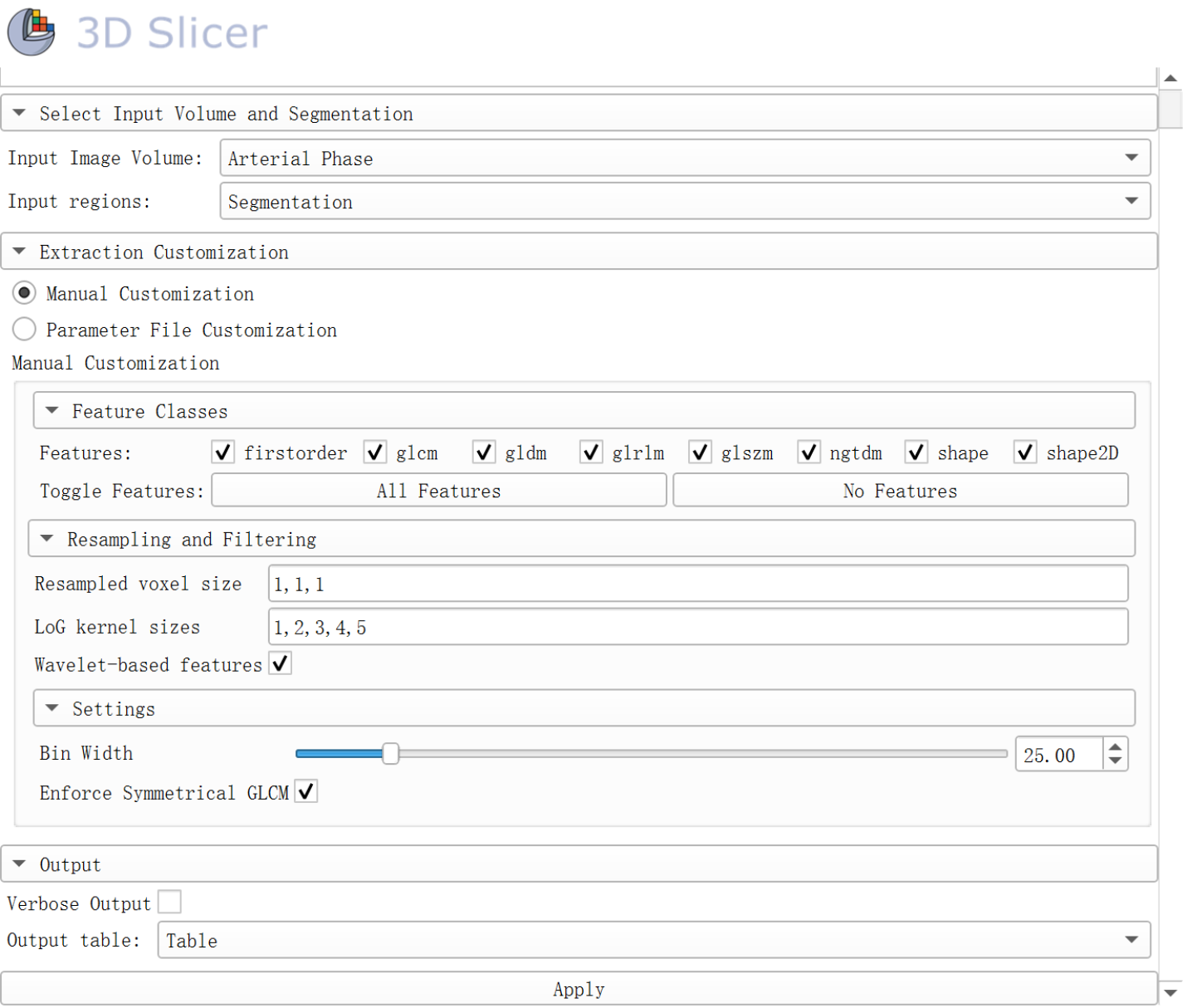

Supplement: Supplementary Figure 1 — Demonstration of the settings for radiomics feature extraction using the SlicerRadiomics extension plugin in the 3D-slicer software. [file Image1.tif]

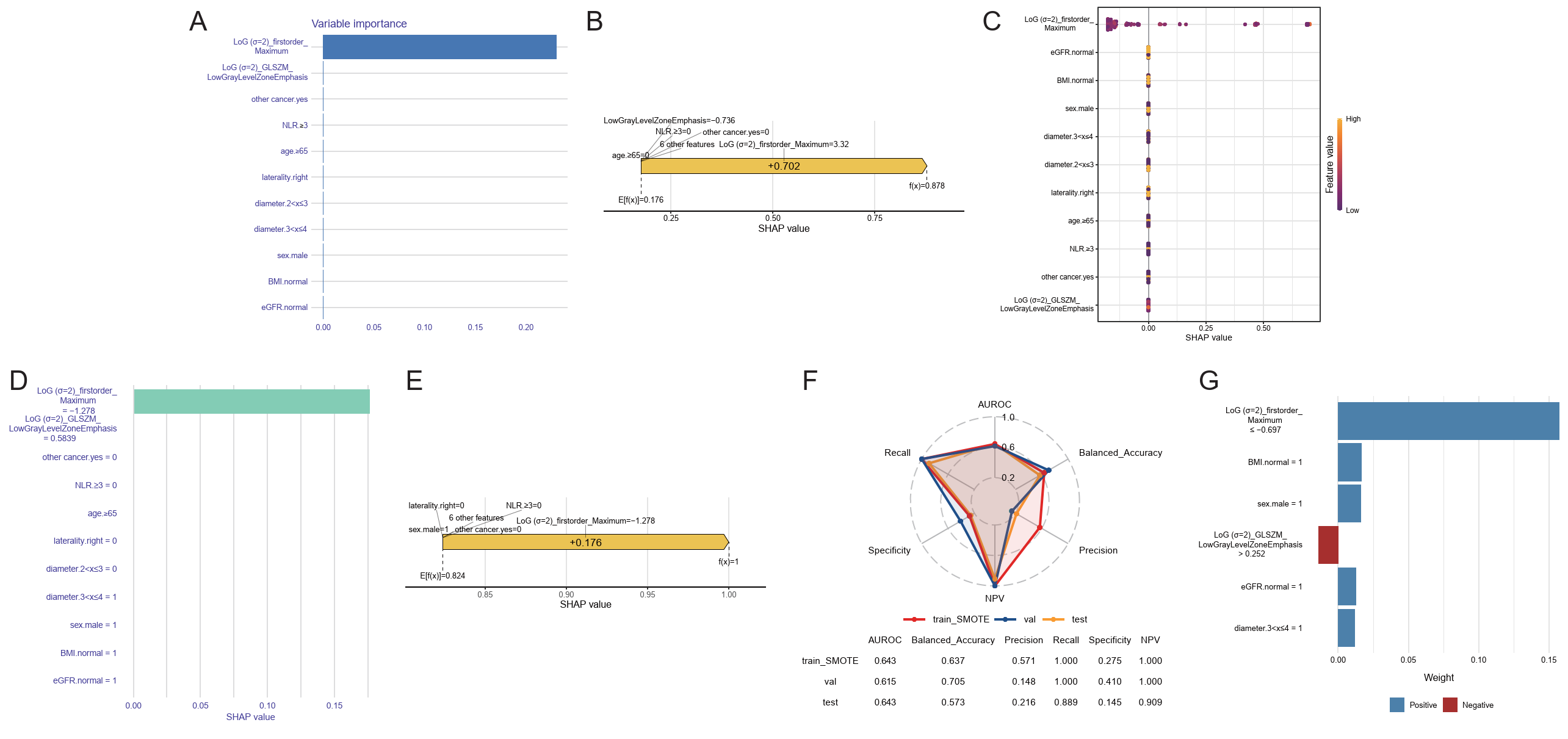

Supplement: Supplementary Figure 2 — Model Interpretability. Global explanations include variable importance (A) and SHAP values (B, C), and local explanations include SHAP values (D, E) and LIME (G). Since variable importance and SHAP only show the importance of the radiomics feature LoG(σ=2)_firstorder_Maximum, only the feature was used for prediction, and the results show that the performance metrics drop significantly compared with before (F). BMI, body mass index; other cancer, history of previous or existing other cancers; NLR, Neutrophil-to-Lymphocyte Ratio; eGFR, estimated Glomerular Filtration Rate; train_SMOTE, train_SMOTE cohort; val, validation cohort; test, test cohort; AUROC, the area under the receiver operating characteristic curve; NPV, Negative Predictive Value; SHAP, Shapley Additive exPlanations; LIME, local interpretable model-agnostic explanations. [file Image2.tif]
