# Supplementary material for: Combining clinical characteristics with CT radiomics to predict Ki67 expression level of small renal mass based on artificial intelligence algorithms
Source: Front Oncol. 2025 Feb 21;15:1541143. doi: 10.3389/fonc.2025.1541143 (PMC11885116; doi:10.3389/fonc.2025.1541143)
Supplement: Supplementary file 4 [file Table1.docx]

**Supplementary Table 1.** Baseline characteristics of high Ki67 expression patients of the primary training cohort and the cohort generated by SMOTE oversampling.

|  | **Train, N=11** | **Train_SMOTE, N=88** | ***P*** |
| --- | --- | --- | --- |
| **Age (y)** |  |  | 1.000 |
| <65 | 7 (63.64%) | 57 (64.77%) |  |
| ≥65 | 4 (36.36%) | 31 (35.23%) |  |
| **Sex** |  |  | 1.000 |
| Female | 3 (27.27%) | 25 (28.41%) |  |
| Male | 8 (72.73%) | 63 (71.59%) |  |
| **BMI** |  |  | 1.000 |
| Normal | 9 (81.82%) | 70 (79.55%) |  |
| Abnormal | 2 (18.18%) | 18 (20.45%) |  |
| **Other cancer** |  |  | 1.000 |
| Yes | 1 (9.09%) | 10 (11.36%) |  |
| No | 10 (90.91%) | 78 (88.64%) |  |
| **Diameter (x)** |  |  |  |
| 0<x≤1 | 1 (9.09%) | 5 (5.68%) | 0.964 |
| 1<x≤2 | 2 (18.18%) | 16 (18.18%) |  |
| 2<x≤3 | 2 (18.18%) | 20 (22.73%) |  |
| 3<x≤4 | 6 (54.55%) | 47 (53.41%) |  |
| **Laterality** |  |  | 1.000 |
| Left | 3 (27.27%) | 26 (29.55%) |  |
| Right | 8 (72.73%) | 62 (70.45%) |  |
| **NLR** |  |  | 1.000 |
| <3 | 8 (72.73%) | 67 (76.14%) |  |
| ≥3 | 3 (27.27%) | 21 (23.86%) |  |
| **eGFR** |  |  | 0.966 |
| Normal | 2 (18.18%) | 21 (23.86%) |  |
| Abnormal | 9 (81.82%) | 67 (76.14%) |  |
| **Ki67 expression** |  |  | - |
| <10% | 11 (100.00%) | 88 (100.00%) |  |
| ≥10% | 0 (0.00%) | 0 (0.00%) |  |

Train, training cohort; train_SMOTE, train_SMOTE cohort; BMI, body mass index; Other cancer, history of previous or existing other cancers; NLR, Neutrophil-to-Lymphocyte Ratio; eGFR, estimated Glomerular Filtration Rate; Normal of BMI and eGFR represents 18.5 ≤ BMI < 25 and eGFR ≥ 90, respectively. Calculation formulas: BMI = weight / (height)^2; NLR = Blood neutrophils / Blood lymphocytes; eGFR = 142 * min(Scr/k,1)^α * max(Scr/k,1)^(-1.200) * 0.9938^age * 1.012[if female], where Scr is serum creatinine, k is 0.7 for females and 0.9 for males, α is -0.241 for females and -0.302 for males, min indicates the minimum of Scr/k or 1, and max indicates the maximum of Scr/k or 1.
