# Supplementary material for: Combining clinical characteristics with CT radiomics to predict Ki67 expression level of small renal mass based on artificial intelligence algorithms
Source: Front Oncol. 2025 Feb 21;15:1541143. doi: 10.3389/fonc.2025.1541143 (PMC11885116; doi:10.3389/fonc.2025.1541143)
Supplement: Supplementary file 5 [file Table2.docx]

**Supplementary Table 2.** Machine learning models used for prediction and the corresponding method in the caret package.

| **Model class** | **Model** | **Method** |
| --- | --- | --- |
| Adaptive Boosting | AdaBoost Classification Trees | adaboost |
| Adaptive Boosting | Bagged AdaBoost | AdaBag |
| Discriminant Analysis | Linear Discriminant Analysis | lda |
| Discriminant Analysis | Linear Distance Weighted Discrimination | dwdLinear |
| Discriminant Analysis | Sparse Linear Discriminant Analysis | sparseLDA |
| Discriminant Analysis | Sparse Distance Weighted Discrimination | sdwd |
| Discriminant Analysis | Stabilized Linear Discriminant Analysis | slda |
| Discriminant Analysis | Distance Weighted Discrimination with Radial Basis Function Kernel | dwdRadial |
| Discriminant Analysis | High Dimensional Discriminant Analysis | hdda |
| Discriminant Analysis | Mixture Discriminant Analysis | mda |
| Discriminant Analysis | Adaptive Mixture Discriminant Analysis | amdai |
| Discriminant Analysis | Penalized Discriminant Analysis | pda |
| Discriminant Analysis | Shrinkage Discriminant Analysis | sda |
| eXtreme Gradient Boosting | eXtreme Gradient Boosting | xgbTree |
| Gaussian Process | Gaussian Process with Polynomial Kernel | gaussprPoly |
| Gaussian Process | Gaussian Process with Radial Basis Function Kernel | gaussprRadial |
| Generalized Linear Model | Generalized Linear Model | glm |
| Generalized Linear Model | Generalized Linear Model with Stepwise Feature Selection | glmStepAIC |
| Generalized Linear Model | Bayesian Generalized Linear Model | bayesglm |
| Generalized Linear Model | Boosted Generalized Linear Model | glmboost |
| Generalized Linear Model | glmnet | glmnet |
| Logistic Regression | Regularized Logistic Regression | regLogistic |
| Logistic Regression | Penalized Logistic Regression | plr |
| Logistic Regression | Boosted Logistic Regression | LogitBoost |
| k-Nearest Neighbors | k-Nearest Neighbors | knn |
| Multivariate Adaptive Regression Spline | Multivariate Adaptive Regression Spline | earth |
| Multivariate Adaptive Regression Spline | Bagged MARS | bagEarth |
| Multivariate Adaptive Regression Spline | Bagged MARS using gCV Pruning | bagEarthGCV |
| Multilayer Perceptron | Multi-Layer Perceptron | mlp |
| Multilayer Perceptron | Multi-Layer Perceptron, with multiple layers | mlpML |
| Multilayer Perceptron | Multilayer Perceptron Network by Stochastic Gradient Descent | mlpSGD |
| Multilayer Perceptron | Multilayer Perceptron Network with Dropout | mlpKerasDropout |
| Multilayer Perceptron | Multilayer Perceptron Network with Weight Decay | mlpKerasDecay |
| Neural Network | Neural Network | nnet |
| Neural Network | Neural Networks with Feature Extraction | pcaNNet |
| Neural Network | Model Averaged Neural Network | avNNet |
| Neural Network | Stacked AutoEncoder Deep Neural Network | dnn |
| Naive Bayes | Naive Bayes | nb |
| Partial Least Squares | Partial Least Squares | pls |
| Partial Least Squares | Partial Least Squares Generalized Linear Models | plsRglm |
| Partial Least Squares | Generalized Partial Least Squares | gpls |
| Partial Least Squares | Sparse Partial Least Squares | spls |
| Tree Model | C5.0 | C5.0 |
| Tree Model | CART | rpart |
| Tree Model | Bagged CART | treebag |
| Tree Model | Conditional Inference Tree | ctree |
| Tree Model | Tree Models from Genetic Algorithms | evtree |
| Tree Model | Boosted Tree | blackboost |
| Tree Model | Tree-Based Ensembles | nodeHarvest |
| Random Forest | Random Forest | ranger |
| Random Forest | Conditional Inference Random Forest | cforest |
| Random Forest | Parallel Random Forest | parRF |
| Random Forest | Regularized Random Forest | RRF |
| Random Forest | Weighted Subspace Random Forest | wsrf |
| Rotation Forest | Rotation Forest | rotationForest |
| Support Vector Machines | Support Vector Machines with Linear Kernel | svmLinear |
| Support Vector Machines | Support Vector Machines with Polynomial Kernel | svmPoly |
| Support Vector Machines | Support Vector Machines with Radial Basis Function Kernel | svmRadial |
| Support Vector Machines | Support Vector Machines with Class Weights | svmRadialWeights |
| Support Vector Machines | Linear Support Vector Machines with Class Weights | svmLinearWeights |
| Adjacent Categories Probability Model | Adjacent Categories Probability Model for Ordinal Data | vglmAdjCat |
| Multinomial Regression | Penalized Multinomial Regression | multinom |
| Ordinal Regression | Penalized Ordinal Regression | ordinalNet |
| Radial Basis Function Network | Radial Basis Function Network | rbfDDA |
| Nearest Shrunken Centroids | Nearest Shrunken Centroids | pam |
| Non-Informative Model | Non-Informative Model | null |
